# Different intraoperative joint laxity patterns do not impact clinical outcomes in robotic‐assisted medial unicompartmental knee replacement with 1‐to‐1 surface reconstruction

**DOI:** 10.1002/ksa.12415

**Published:** 2024-08-08

**Authors:** Matteo Innocenti, Filippo Leggieri, Carlo Theus‐Steinman, Joaquin Moya‐Angeler, Bernhard Christen, Tilman Calliess

**Affiliations:** ^1^ Department of Clinical Orthopedics, A.O.U. Careggi CTO University of Florence Florence Italy; ^2^ Articon Spezialpraxis für Gelenkchirurgie Bern Switzerland; ^3^ Department of Orthopaedic Surgery Hospital General Universitario Reina Sofia Murcia Spain

**Keywords:** gap balance, HKA correlation, knee balance, medial unicompartmental arthroplasty, soft tissue laxity

## Abstract

**Purpose:**

Robotic‐assisted technology in medial unicompartmental knee arthroplasty (mUKA) allows for customized adjustments of joint laxity through virtual preoperative component positioning before bone preparation. Nevertheless, the optimal balancing curve has yet to be delineated. This study sought to investigate if varying intraoperative knee laxity patterns had any impact on postoperative patient outcomes.

**Materials and Methods:**

A retrospective analysis was conducted on prospectively collected data from 326 fixed‐bearing RAUKA procedures performed between 2018 and 2022 with a minimum 2‐year follow‐up. Patients were categorized into three cohorts based on intraoperative joint laxity patterns (millimetres of joint gap during valgus stress) imparted at 20°, 60°, 90° and 120° of knee flexion: cohort 1 < +0.5 mm (tight); cohort 2 between 0.6 and 1.9 mm (physiologic); cohort 3 > 2 mm (loose). Wilcoxon and Kruskal–Wallis tests were conducted to assess patient‐reported outcome measure (PROM) improvements and preoperative and postoperative differences across the cohorts. A Spearman's test evaluated the correlation between knee balance at all degrees of flexion and preoperative and postoperative HKA.

**Results:**

No differences in preoperative and postoperative PROMs were identified across the cohorts (*p* > 0.05). All three cohorts with different joint laxity patterns showed a significant improvement in the postoperative PROMS (*p* < 0.05). The preoperative or postoperative limb alignment did not significantly affect clinical outcomes relative to different laxity patterns.

**Conclusion:**

No differences were found in the outcomes across different joint laxity patterns in robotic‐assisted medial UKA using fixed‐bearing mUKAs. There was no evident advantage for maintaining a closer to physiologic laxity compared to tighter or looser balancing.

**Level of Evidence:**

Level III, therapeutic study.

**Abbreviations**
ADL: advance daily living
CPAK: coronal plane alignment of the knee
CR: cruciate retaining
EQ‐5D: European Quality of Life 5 Dimensions 5 Level Version
FCA: femoral component coronal angle
HKA: hip–knee–ankle
KOOS: Knee Injury and Osteoarthritis Outcome Score
KSS: Knee Society Score
MPS: MAKO Product Specialist
mUKA: medial unicompartmental knee arthroplasty
OKS: Oxford Knee Score
P: pain
PROM: patient‐reported outcome measure
PS: posterior stabilized
QoL: quality of living
RA: robotic assisted
ROM: range of motion
S: symptoms
STA: sagittal tibial angle
TCA: tibial component coronal angle
TKA: total knee arthroplasty
UKA: unicompartmental knee arthroplasty

## INTRODUCTION

The success of resurfacing the medial knee joint compartment and restoring the normal knee kinematics through the preservation of both cruciate and collateral ligaments with a medial unicompartmental knee arthroplasty (mUKA) cannot overlook a proper balancing of the soft tissues [4, 7, 15, 23, 29]. Accurate intra‐operative assessment is required to ascertain the state of soft tissue balance and to guide the surgical intervention towards achieving the desired balance of the knee. Computer‐assisted and robotic‐assisted surgery offers useful tools for such purposes [11, 12, 16, 17].

Negligence about soft tissue tension may lead to stiffness or instability and poly wear, which are widely acknowledged contributors to complications and failures after mUKA [18, 28]. Such definitive assurance cannot be unequivocally extended to patient‐reported satisfaction and patient‐reported outcome measures (PROMs) resulting from diverse patterns of soft tissue balancing across various degrees of knee flexion. The correlation between soft tissue balancing and clinical outcomes remains poorly elucidated, as does the characterization of the ‘ideal’ balancing curve.

Matsuzaki et al. found a correlation between the intraoperative joint component gap, joint laxity in mid‐flexion and postoperative knee flexion in mUKA, suggesting that the postoperative range of motion (ROM) is influenced by intraoperative soft tissue balancing. However, no additional correlations with postoperative PROMs were documented [22].

To the current extent of knowledge, no studies have systematically explored the relationship between patient outcomes and the achieved joint laxity combined with overall limb alignment following mUKA.

The objective of this study was to ascertain if different patterns of intraoperative soft tissue laxity, applied across distinct degrees of knee flexion, could have had a different impact on postoperative PROMs following mUKA. It was hypothesized that either overtightening or undertightening the medial compartment, with precise intraoperative quantification facilitated by robot‐assisted surgical techniques, could affect the postoperative outcomes for patients undergoing mUKA.

## MATERIALS AND METHODS

Prospectively collected data of 378 mUKAs performed at a single institution between October 2018 and March 2022 were retrospectively reviewed. All cases were operated by two highly trained knee surgeons in robotic‐assisted surgery.

The study included all patients who had a comprehensive data set encompassing preoperative, intraoperative and postoperative outpatient clinical and radiological evaluations. Exclusion criteria were patients who withheld informed consent for participation in the study, those with less than 24 months of follow‐up and individuals for whom intraoperative soft tissue laxity data were not documented. Consequently, 326 knees were included in the study.

Three cohorts were identified according to the soft tissue laxity intraoperatively (millimetres of laxity) imparted at 20°, 60°, 90° and 120° of knee flexion measured with the image‐based robotic system at the end of the surgery. The cut‐offs for each cohort were set as follows: cohort 1 included values below +0.5 mm (tight); cohort 2 included values between 0.6 and 1.9 mm (physiologic balance); cohort 3 included values above 2 mm (loose).

The research was conducted in adherence to the ethical guidelines set forth by the Declaration of Helsinki and its later amendments. All participants provided informed consent for the prescribed treatment protocol, the surgical procedure and the postoperative rehabilitation and follow‐up strategy. Additionally, informed consent was obtained from all patients for the collection of their data and its anonymized utilization for scientific research purposes.

### Surgical technique

The preferred alignment, implant size and positioning strategies were customized for each patient by the surgeon using MAKO® software to closely reconstruct the native prearthritic joint surface with the implants following a surgical technique previously described [9]. The same fixed‐bearing metal‐backed cemented unicompartmental knee prosthesis was implanted (RESTORIS MCK partial knee; Stryker). The detailed surgical protocol applied to every patient is described in the Supporting Information document.

### Intraoperative soft tissue balance assessment

**Figure 1:**
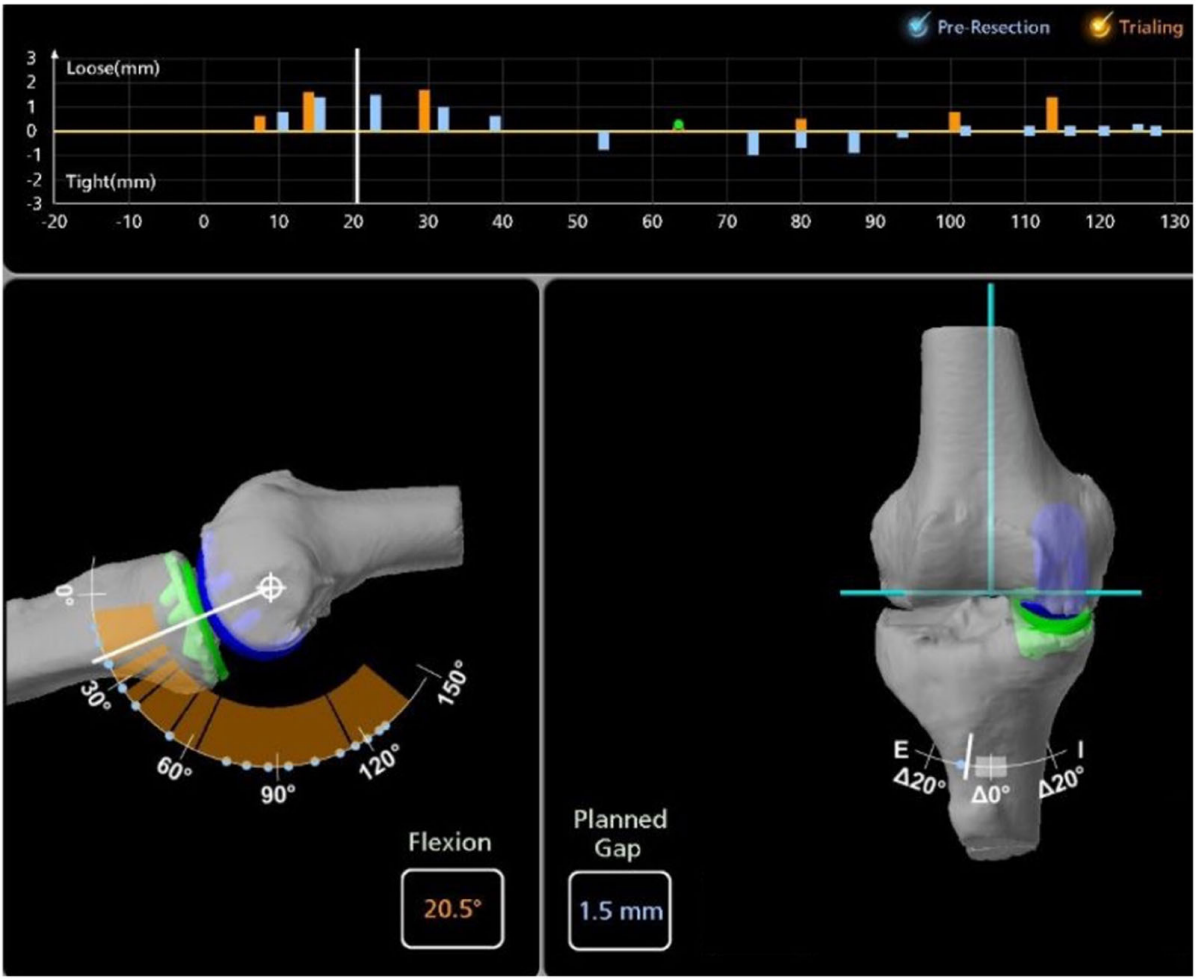
Three patient groups were classified based on the degree of soft tissue laxity patterns measured intraoperatively at a knee flexion angle of 20°, 60°, 90° and 120° using the image‐based robotic system.

To balance the flexion‐extension gaps throughout the knee's entire ROM and obtain femorotibial tracking data, the knee was positioned at multiple flexion angles while applying valgus stress and slowly taking the limb through a ROM at different degrees such as between 10–30° of flexion, 60–90° of flexion and 100–120° of deep‐flexion. When all positions were captured, the gap laxity graph displayed the predicted joint gaps (mm) for the captured flexion poses. For the purposes of this article, the maximum gap laxity in millimetres at 20°, 60°, 90° and 120° of flexion was recorded (Figure 1).

### Population and follow‐up

Demographic characteristics such as age at the time of the surgery, gender, body mass index, affected side, diagnosis, surgery date and outpatient follow‐up data date were recorded.

The hip–knee–ankle (HKA) angle, the mechanical lateral distal femur angle and the mechanical medial proximal tibia angle were calculated on the preoperative long film standing X‐rays. The Oxford Knee Score (OKS) [5], the Knee Injury and Osteoarthritis Outcome Score (KOOS) [26], the Knee Society Score (KSS) [10] and the European Quality of Life 5 Dimensions 5 Level Version (EQ‐5D) [8] score were assessed preoperatively. At 2‐year postoperative outpatient follow‐up, the HKA, the femoral component coronal angle and the tibial component coronal angle were calculated on full‐leg‐standing X‐rays and the same previously reported PROMs were recorded [20].

Additionally, any intraoperative and postoperative complications were registered.

### Statistical analysis

Statistical analyses were performed using SPSS software (IBM Corp. Released 2017. IBM SPSS Statistics for Windows, version 25.0). Data are expressed as mean ± standard deviation (range, max–min) unless otherwise indicated. The level of statistical significance was set at *p* < 0.05. A Shapiro–Wilk test was performed to test for normality. To assess for missing data, a stepwise approach was performed throughout the nonparametric tests. A Wilcoxon rank test was conducted to detect any improvements in the postoperative PROMs across each cohort for every degree of flexion. The Kruskal–Wallis test was used to analyze any differences in preoperative and postoperative clinical scores across the cohorts. A Spearman correlation test was performed to assess the correlation between the knee balance at all degrees of flexion; the preoperative and the postoperative HKA measurements were performed. An a priori power analysis was conducted to determine the appropriate sample size for the study. An analysis of variance (fixed effects, omnibus, one‐way) test was selected to compare the means of three independent groups using G*Power 3.1 software. The power analysis utilized an effect size (*f*) of 0.5, with an *α* level set at 0.05 and a desired power (1 − *β*) of 0.8. Given these parameters and the inclusion of three groups, it was determined that a total sample size of 42 participants would be required to achieve the desired power level.

## RESULTS

**Figure 2:**
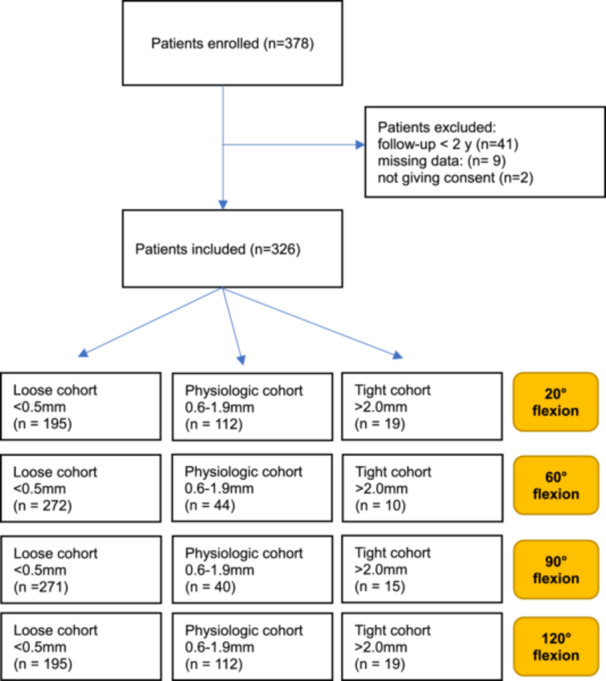
Flowchart of the final included population for all degrees of knee flexion.

A flowchart representing the final included population distribution across the cohorts is shown in Figure 2. Patients' baselines and characteristics are shown in Table 1, including the mean value of the knee joint maximum laxity (in mm) registered at 20°, 60°, 90° and 120° of flexion. The distribution of the different joint laxity patterns across the cohorts at all degrees of flexion angles is shown in Table 2.

**Table 1:** Patients' baseline and characteristics.

	Results	SD	Min	Max
Patients (*n*)	326			
Mean BMI (kg/m²)	28.42	14.21	17	269
Right side (*n* [%])	151 [46.3]			
Male sex (*n* [%])	145 [44.5]			
Mean age at surgery (year)	66.35	8.89	39	87
Mean HKA (°)	175.29	4.85	165	192
Mean LDFA (°)	87.41	8.60	81	95
Mean MPTA (°)	85.88	8.58	80	94
Mean postoperative HKA (°)	178.53	2.78	169	188
Mean postoperative FCA (°)	88.19	2.10	83	95
Mean postoperative TCA (°)	86.96	2.41	81	96
Mean balance at 20°	0.56	0.67	−0.4	0.5
Mean balance at 60°	0.24	0.69	−1.4	4.5
Mean balance at 90°	0.24	0.73	−2.2	5.2
Mean balance at 120°	0.27	0.78	−2	4

Abbreviations: BMI, body mass index; FCA, femoral component coronal angle; HKA, hip–knee–ankle; SD, standard deviation; TCA, tibial component coronal angle.

**Table 2:** Distribution of the different joint laxity pattern across cohorts at all degrees of flexion angles.

	20°			60°			90°			120°		
	Mean	Min	Max	Mean	Min	Max	Mean	Min	Max	Mean	Min	Max
Loose	0.1 ± 0.20	−0.4	0.5	0.02 ± 0.25	−1.4	0.5	0.0 ± 0.29	−2.2	0.5	−0.02 ± 0.34	−2	0.5
Physiologic	1.06 ± 0.35	0.6	1.9	0.9 ± 0.28	0.6	1.7	1.0 ± 0.35	0.6	1.9	0.95 ± 2.6	06	1.7
Tight	2.27 ± 0.38	2	3.3	3.32 ± 0.95	2	4.5	2.98 ± 1	2	5.2	2.7 ± 0.68	2	4

The Shapiro–Wilk test revealed a mix of normal and nonnormal distributions among the variables. Given this variability in the data distribution, nonparametric statistical methods were subsequently employed to ensure a robust and accurate analysis.

No significant differences across the cohorts for preoperative and postoperative KSS, OKS, EQ‐5D and KOOS scoring results were found at all degrees of flexion as shown in Table 3.

**Table 3:** Kruskal–Wallis test for pre‐ and postoperative PROMs across the cohorts at all degrees of flexion.

	Preoperative								Postoperative							
	20°		60°		90°		120°		20°		60°		90°		120°	
	*H*	Sig.	*H*	Sig.	*H*	Sig.	*H*	Sig.	*H*	Sig.	*H*	Sig.	*H*	Sig.	*H*	Sig.
KSS	5.30	0.07	2.64	0.26	0.54	0.76	0.39	0.82	4.80	0.09	1.87	0.39	0.17	0.91	1.01	0.60
OKS	5.87	0.53	1.53	0.46	1.64	0.44	0.33	0.84	3.36	0.18	0.83	0.65	0.45	0.79	0.50	0.77
KOOS	2.77	0.25	1.54	0.46	1.54	0.46	0.13	0.93	4.08	0.13	2.26	0.32	2.31	0.31	0.69	0.70
EQ‐5D	3.258	0.19	1.01	0.60	0.37	0.828	2.21	0.33	0.43	0.80	2.40	0.31	1.85	0.39	2.8	0.24

*Note*: Sig., statistical significance at *p* < 0.005.

Abbreviations: EQ‐5D, European Quality of Life 5 Dimensions 5 Level Version; KOOS, Knee Injury and Osteoarthritis Outcome Score; KSS, Knee Society Score; OKS, Oxford Knee Score; PROM, patient‐reported outcome measure.

Significant improvements were found postoperatively across the cohorts at all degrees of flexion for KOOS, OKS, KSS and EQ‐5D scores as shown in Table 4.

**Table 4:** Wilcoxon test signed rank pairwise test for PROMs across the cohorts at the final follow‐up.

	Loose								Physiologic								Thigh							
	20°		60°		90°		120°		20°		60°		90°		120°		20°		60°		90°		120°	
	*Z*	Sig.	*Z*	Sig.	*Z*	Sig.	*Z*	Sig.	*Z*	Sig.	*Z*	Sig.	*Z*	Sig.	*Z*	Sig.	*Z*	Sig.	*Z*	Sig.	*Z*	Sig.	*Z*	Sig.
KSS	11.63	<0.01*	13.78	<0.01*	13.86	<0.01*	13.48	<0.01*	8.93	<0.01*	5.64	<0.01*	5.23	<0.01*	5.84	<0.01*	3.72	<0.01*	2.66	0.01*	3.06	<0.01*	3.62	<0.01
OKS	11.64	<0.01*	13.61	<0.01*	13.73	<0.01*	13.34	<0.01*	8.71	<0.01*	5.64	<0.01*	5.13	<0.01*	5.83	<0.01*	3.43	<0.01*	2.32	0.02*	2.82	<0.01*	3.36	<0.01
KOOS	10.39	<0.01*	12.49	<0.01*	12.61	<0.01*	12.16	<0.01*	8.59	<0.01*	5.64	<0.01*	5.15	<0.01*	5.84	<0.01*	3.51	<0.01*	2.52	0.01*	2.93	<0.01*	3.51	<0.01
EQ‐5D	9.52	<0.01*	11.43	<0.01*	11.66	<0.01*	11.27	<0.01*	7.90	<0.01*	5.27	<0.01*	4.56	<0.01*	5.16	<0.01*	3.36	<0.01*	2.52	0.01*	2.80	<0.01*	3.30	<0.01

*Note*: Sig., statistical significance at *p* < 0.005.

Abbreviations: EQ‐5D, European Quality of Life 5 Dimensions 5 Level Version; KOOS, Knee Injury and Osteoarthritis Outcome Score; KSS, Knee Society Score; OKS, Oxford Knee Score; PROM, patient‐reported outcome measure.

* Denotes statistical significance was reached.

The correlation tests revealed no significant relationships between postoperative HKA angles and knee laxity at different degrees of knee flexion, as illustrated in Table 5. Similarly, no correlations were found between preoperative HKA angles and knee laxity at 20° of knee flexion. Intraoperative complications counted for two femoral array dislocations and one malfunction in the robotic burr needing an exchange. No consequences followed but an increase in the operative time. No complication was registered during the hospital stay or postoperatively. No revision or reintervention was needed within the 2‐year follow‐up across the cohorts.

**Table 5:** Spearman's correlation coefficients between HKA and knee laxity across the cohorts at all degrees of flexion.

Joint laxity	Preoperative HKA	Postoperative HKA
20°		
*ρ*	0.04	0.06
Sig. (two‐tailed)	0.40	0.24
60°		
*ρ*	0.02	0.09
Sig. (two‐tailed)	0.68	0.11
90°		
*ρ*	0.02	0.09
Sig. (two‐tailed)	0.68	0.11
120°		
*ρ*	0.02	0.09
Sig. (two‐tailed)	0.68	0.11

*Note*: Sig., statistical significance at *p* < 0.005.

## DISCUSSION

The most important finding of the study was that different intraoperative soft tissue laxity patterns, observed at multiple degrees of knee flexion, did not exert a significant impact on postoperative PROMs following mUKA. This suggests that the clinical success of mUKA, demonstrated by the significant postoperative improvements in KOOS, OKS, KSS and EQ‐5D scores across all the cohorts, remains robust despite the variability in joint laxity patterns observed during surgery [19, 31]. Despite these findings, the present study was unable to determine an ideal joint laxity pattern for robotic‐assisted UKA that would optimize patient outcomes.

This is the first study aiming to determine whether different intraoperative soft tissue laxity patterns imparted at different degrees of knee flexion of the knee could have any consequence on postoperative PROMs after mUKA. Indeed, previous reports that have investigated whether different intraoperative knee laxity patterns could influence PROMs and ROM were focussed on total knee arthroplasty (TKA) and not on UKA.

Matsumoto et al. reported that the intraoperative joint component gap and the ligament balance measurements did not correlate between intraoperative and postoperative clinical outcomes in CR and PS TKA at the 5‐year follow‐up using a tensor during flexion [21]. McDessi et al. reported that the intraoperatively use of pressure sensors compared to manual balancing did not lead to a greater improvement in KOOS or in other knee‐specific or general health outcomes scores at 2 years postoperatively. They also found no differences in the postoperative outcomes when quantitatively well‐balanced knees were compared to knees with mild, moderate or severe imbalance. Similarly, Sarpong et al. and Wood et al., through RCTs, demonstrated that the use of a sensor‐based balancing device for soft tissue balancing in TKA yielded no significant advantages in postoperative ROM, PROMs or clinical outcomes [27, 30]. In a recent systematic review carried out by Batalier et al., it was corroborated that intraoperative sensor technology did not demonstrate a correlation with enhanced clinical outcomes following TKA [2]. Ultimately, inferior postoperative outcomes and lower levels of satisfaction were documented in cases of intraoperative medial gaps with residual laxity. Conversely, patients exhibiting a slightly more lax lateral compartment in flexion in comparison to the medial compartment following TKA tend to demonstrate improved clinical scores [1, 3, 13, 14, 25].

Another finding from the current investigation is the existence of a noncorrelative relationship between the preoperative and postoperative HKA angles and the resulting laxity pattern following mUKA.

These results are consistent with previous data in the literature. Nakano et al. aimed to find a correlation between preoperative varus deformity of the knee and the intraoperative joint gap in mUKAs [24]. While it was found, a positive correlation between the severity of preoperative varus deformity and the preosteotomy gap in extension, no correlation was proven between the preoperative HKA and the preosteotomy gap at flexion. However, Ge et al. found that the interprosthesis pressure, intraoperatively assessed through sensor technology at 0° and 20°, had a positive correlation with postoperative HKA following Oxford mUKAs. This different finding could be due to the smaller population size included compared to the one of the current study and the different type of implants enroled [6].

The study has some limitations: soft tissue balancing values showed low dispersion indexes. As the population was divided into three cohorts, the distribution of the laxity values within each cohort appeared to range in proximity to the mean of the overall population's values. This might indicate the effect of a small sample size, which could have underpowered the statistical analysis, thereby failing to detect differences in postoperative outcomes. However, it is also possible that such low dispersion indexes do not represent a distortion due to the small sample size. Support to this observation is the fact that intraoperative balance was measured and imparted by two experienced surgeons with the robotic assistance. Thus, the absence of any differences in the joint laxity pattern not translating clearly and perceivably to the knee functionality demonstrates no to be a clinically relevant data.

## CONCLUSION

This study observed significant postoperative PROM improvements after mUKA regardless different intraoperative medial joint laxity patterns. Variations in intraoperative soft tissue balance at all degrees of flexion, whether tighter or looser, had no significant impact on postoperative clinical outcomes. Moreover, a noncorrelative relationship between postoperative HKA and gap laxity of the knee was found.

## AUTHOR CONTRIBUTIONS

Tilman Calliess, Bernhard Christen and Matteo Innocenti proposed the concept of the study and designed the study. Filippo Leggieri and Carlo Theus‐Steinman have drafted the original version of the work and performed the analysis and interpretation of data. Matteo Innocenti, Tilman Calliess and Joaquin Moya‐Angeler revised the various versions of the study. Filippo Leggieri, Bernhard Christen and Carlo Theus‐Steinman participated in the bibliographic collection, in the data check and in the production of the tables and the images and reviewed the article. Each author has read and approved the final version of the manuscript and has agreed to be personally accountable for the author's own contributions and for ensuring that questions related to the accuracy or integrity of any part of the work, even ones in which the author was not personally involved, are appropriately investigated, resolved and documented in the literature. The authors have no funding to report.

## CONFLICT OF INTEREST STATEMENT

The authors declare no conflict of interest related to the subject of this study. Tilman Calliess and Bernhard Christen are paid consultants for education by STRIKER.

## ETHICS STATEMENT

The authors certify that all investigations were conducted in conformity with ethical standards of the institutional and national research committee and with the 1964 Helsinki Declaration and its later amendments. All patients have given their informed consent for participation.

## Supporting information

Supporting information.

## Data Availability

The data supporting the findings of this study can be obtained upon request from the corresponding author, F. L. These data are not publicly accessible due to restrictions related to compromising the privacy of research participants.
