# Supplementary material for: Different intraoperative joint laxity patterns do not impact clinical outcomes in robotic‐assisted medial unicompartmental knee replacement with 1‐to‐1 surface reconstruction
Source: Knee Surg Sports Traumatol Arthrosc. 2024 Aug 8;32(12):3299–307. doi: 10.1002/ksa.12415 (PMC11605022; doi:10.1002/ksa.12415)
Supplement: Supplementary file 1 — Supporting information. [file KSA-32-3299-s001.docx]

**Surgical technique**

Before surgery, a standard X-rays protocol for UKA was performed for every patient along with a CT scan with the specific radiographic protocol required for preoperative templating. A Preoperative CT-scan of the hip, knee, and ankle was conducted following the standard MAKOPlasty protocol and uploaded to STRYKER proprietary platform (Stryker, Mahwah, USA). Based on this scan an individual knee model was segmented by the on-site MAKO Product Specialist (MPS) and used for the primary surgical planning according to the STRYKER standard protocol. The preferred alignment, implant size, and positioning strategies were customized for each patient by the surgeon just before the surgery in the operating room using MAKO® software to closely reconstruct the native prearthritic joint surface anatomy with the implants a surgical technique previously described[13]

All surgical procedures were performed by using the MAKO® robotic assistance (Stryker®, Mahwah, USA) with the patient in a supine position, and a tourniquet was routinely inflated before making the skin incision. Intravenous antibiotic prophylaxis was administered following the hospital protocol. A midline incision was made, and the knee joint was exposed using a mini medial-parapatellar arthrotomy as the preferred approach for every case. The cruciate ligaments were examined to confirm suitability for mUKA. The same fixed-bearing metal-backed cemented unicompartmental knee prosthesis was implanted (RESTORIS MCK partial knee, Stryker, Mahwah, USA) with the assistance of the MAKO Robotic system. A classical workflow for a MAKO robotic-assisted surgery was used to position the arrays and to register and match the bone anatomy.

Kinematic data about Range of Motion (ROM) and soft tissue laxity were recorded. After osteophytes removal, the ROM and the kinematic data of the knee were acquired by applying valgus stress to reduce the arthritic deformity. The prosthesis is positioned to reconstruct the native surface as best as possible in all three plains and to obtain the best femoro-tibial tracking through the whole range of motion of the knee. By applying those concepts an individual alignment is therefore reached for every single patient (Figure 1). Once bone resection was completed, trial implants were tested with robotic assistance feedback, and final implants were then cemented.

All patients followed the same post-operative physical therapy protocol with free active/passive knee mobilization, quad strength exercises, and weight-bearing as tolerated from the first postoperative day. All patients were discharged home from the hospital.

**
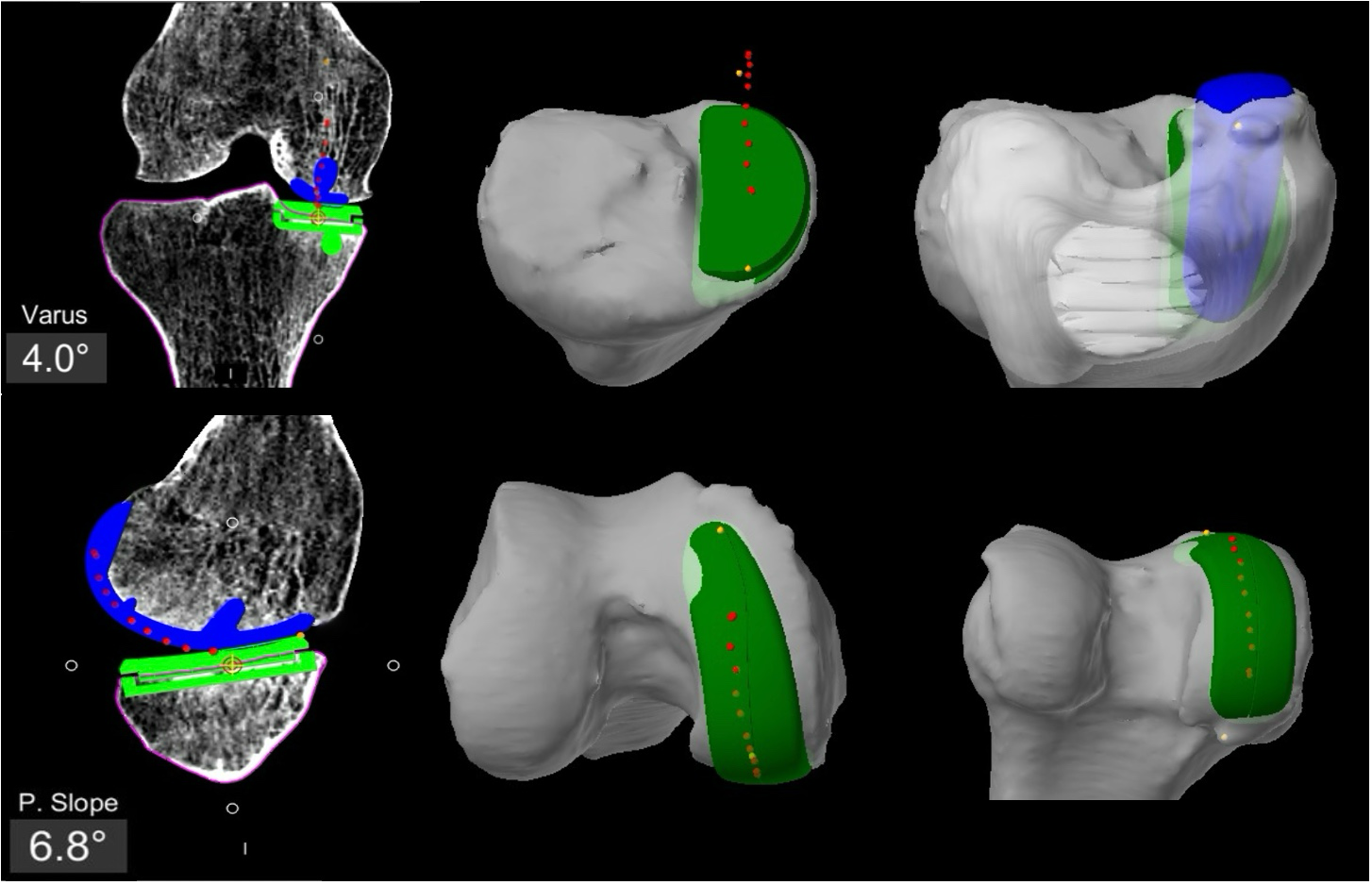
**

**Figure 1.** Assisted by robotic technology, the goal was to attain a personalized alignment tailored to each patient.
